# The Impact of Teammates’ Online Reputations on Physicians’ Online Appointment Numbers: A Social Interdependency Perspective

**DOI:** 10.3390/healthcare8040509

**Published:** 2020-11-23

**Authors:** Jingfang Liu, Xin Zhang, Jun Kong, Liangyu Wu

**Affiliations:** School of Management, Shanghai University, Shanghai 200444, China; jingfangliu@shu.edu.cn (J.L.); charlottek@shu.edu.cn (J.K.); skyrim@shu.edu.cn (L.W.)

**Keywords:** online medical team, online reputation, appointment numbers, leader, teammates, inverted U-shaped relationship, competition, cooperation, social interdependence theory

## Abstract

Online medical team is an emerging online medical model in which patients can choose a doctor to register and consult. A doctor’s reputation cannot be ignored. It is worth studying how that online reputation affects the focal doctor’s appointment numbers on the online medical team. Based on the online reputation mechanism and social interdependence theory, this study empirically studied the impact of the focal doctor’s own reputation and other teammates’ reputation on his/her appointment numbers. Our data include 31,143 doctors from 6103 online expert teams of Guahao.com. The results indicate that for a leader doctor, his/her appointment numbers are not related to his/her own reputation, and there was an inverted U-shaped relationship with the ordinary doctors’ reputations on the team. For an ordinary doctor, his/her appointment numbers were positively correlated with his/her own reputation and positively correlated with his/her leader’s reputation and there was an inverted U-shaped relationship with the other ordinary doctors’ reputations. The research showed that there is a positive spillover effect on the team leader’s reputation. There are two relationships between team doctors: competition and cooperation. This study provides guidance for the leader to select team members and the ordinary doctor to select a team.

## 1. Introduction

The coordination and delivery of safe, high-quality care require reliable teamwork and collaboration across organizational, disciplinary, technical, and cultural boundaries [1,2]. Offline medical teams are very common in Western countries. Effective team cooperation provides safe and effective care for all levels of the medical system [3,4]. In most developed countries, team medicine has become a standard of diagnosis and treatment. Both the leading family doctors and the famous Mayo Clinic work in doctor-collaboration teams [5]. The importance of teamwork is also evident in the response to COVID-19 [6]. Team medicine is helpful for both patients and doctors. For patients, team medical cooperative treatment can effectively improve the diagnosis and treatment ability and efficiency of complex diseases, provide comprehensive nursing and rehabilitation design for patients, and make each patient’s treatment more personalized [7,8]. For the team’s medical staff, it is necessary to change their focus from the concept of emergency to long-term disease prevention and health care. They can learn new technologies and make new breakthroughs in the diagnosis and treatment of each patient and require the medical staff to focus on their professional fields [9,10]. On the team, young doctors can also obtain better training and experience. With the development of the "Internet + healthcare", online medical teams have begun to appear on online medical service platforms in China. China’s haodf.com and guahao.com have set up expert teams. The expert teams are led by brand doctors, joined by ordinary doctors and young doctors, and collaboration teams are established to jointly diagnose and treat patients. The emergence of online medical teams can alleviate the problem that senior experts have no time for branding, and young doctors and grassroots doctors have time but no brand, so that doctors can better allocate time, share experience, and share brands. This will make the distribution of valuable medical resources more reasonable. Online medical teams are a new type of virtual team, so it is necessary to study and analyze them.

As the basis for online transactions, the online reputation mechanism provides an important basis for consumers’ purchase decisions [11,12]. Online reputation is a kind of public praise formed by the evaluation feedback of purchased goods or services after consumers consume online [13,14]. In the past, many scholars have studied online reputation based on online markets such as eBay, Taobao, Yelp, Uber, and Airbnb [15,16,17]. Medical and health services are a typical trust product [18]. Because of information asymmetry, it is difficult for patients to judge the medical service level of doctors. At this time, doctors know their own medical level, but patients do not know the real medical level of doctors before they receive the doctor’s services. So, the patient can only judge the doctor’s medical level through the information on the doctor’s website. At this time, a doctor’s online reputation is particularly important. As a signal of a doctor’s medical service level, the doctor’s online reputation provides a decision-making basis for patients to register online [19,20,21]. Many studies have confirmed the influence of brand on the sales volume of goods or the purchase intention of consumers [22]. In a medical team, the team leader is usually a doctor with high status, whose reputation is equivalent to the brand of the team. Therefore, the reputation of the leader of the team conveys the image of the team to the patients, provides an important basis for the patients to infer the medical level of the team, and reduces the perceived risk and information cost of the patients.

Interdependence and social classification are the foundation of team formation [23]. Koffka demonstrated that the team is a dynamic whole, with different forms and degrees of dependence among members [24]. Lewin proposed that the essence of a team is the interdependence among members of the team. The state change in any member or subteam in a team will affect the state of other members or subteams [25]. According to McGrath, a team is a social collection of existing mutual understanding and potential interactions between members [26]. When the results of individuals are influenced by their own and others’ behaviors, social interdependence is produced [27]. Morton Deutsch put forward the theory of social interdependence in 1949.There are two types of social interdependence: positive (when the individual’s behavior promotes the realization of common goals) and negative (when the individual’s behavior hinders the realization of another’s goals) [28]. When there is a positive-positive relationship between the goals and means of different individuals, there is a cooperative relationship between them, such as the relationship between football team players [29]. Under the condition of task interdependence and resource interdependence, students more easily achieve better results than students who study alone [30]. When there is a negative dependence on the target means of different individuals, there is a competitive relationship between them, such as boxing. When a person needs the resources of other team members but has no common goal (that is, he or she does not share resources with others, but only obtains resources from others), the result will affect another’s productivity [28]. Colasante et al. found that intrateam competition can significantly reduce free-riding behavior [31].

In the field of health care, few people study the relationship between doctors working in the same organization. If an organization has a good reputation, all its members are considered to have a good reputation [32]. Wu et al. found that there are both cooperative relationships and competitive relationships between doctors and colleagues [33]. From the perspective of "cooperation", the reputations of a doctor and all his or her colleagues form the reputation of his or her team. When the reputation of the team is high, the team can attract more patients. At this time, the reputations of the doctor’s colleagues has a positive impact on him or her. From the perspective of "competition", when patients register online, they will select the doctor of the expert team according to the doctor’s online reputation and other signals. At this time, if the doctor’s colleagues have a high online reputation, the doctor’s colleagues are more likely to be selected. At this point, a colleague’s online reputation has a negative impact on the doctor. 

Therefore, it is meaningful to study the impact of teammates’ online reputations in the online medical team on the focal doctor’s appointment numbers.

Since there are two types of doctors in an online medical team, this study divided the doctors in an online medical team into two types, leader doctor and ordinary doctor, and conducted the study separately. The team is led by the leader doctor, and other doctors can join. The leader doctor is often a more senior doctor. Ordinary doctors are the team members other than the leader doctor. Most of them are young doctors and doctors with less seniority. This study mainly solves the following problems:

When the leader acts as the focal doctor:

Research question1: How does the focal doctor’s reputation affect his/her own appointment numbers in the online medical team?

Research question12: How does the reputation of ordinary doctors of the online medical team affect the focal doctor’s appointment numbers?

When the ordinary doctor acts as the focal doctor:

Research question11: How does the focal doctor’s reputation affect his/her own appointment numbers in the online medical team?

Research question12: How does his/her leader’ reputation affect the focal doctor’s appointment numbers?

Research question13: How does the reputation of other ordinary doctors of the online medical team affect the focal doctor’s appointment numbers?

In this study, we tested the research hypotheses using the data collected from Guahao.com. Guahao.com is one of the main online medical service platforms in China focusing on registration services. Guahao.com created a team medical model. A team consists of a well-known team leader doctor and several ordinary doctors. Patients can first select an expert team and then select a doctor from the expert team to register, consult, or purchase other services. 

In August 2019, we collected data from 31,143 doctors from 6103 online expert teams of Guahao.com. Each dataset included the doctor’s appointment numbers, title, department, hospital and city, review numbers, likes, scores, and team size. Then, we used an econometric method to verify our research hypothesis.

## 2. Materials and Methods

### 2.1. Research Model and Hypotheses Development

The purpose of this study was to investigate the impact of the focal doctor’s own reputation and his/her teammates’ reputations of an online medical team on the focal doctor’s appointment numbers. In our research context, there were two types of doctors in an online medical team, leader and ordinary doctors. Therefore, we separated the leader and ordinary doctors to establish different research models.

#### 2.1.1. Hypotheses of Leaders

**Figure 1:**
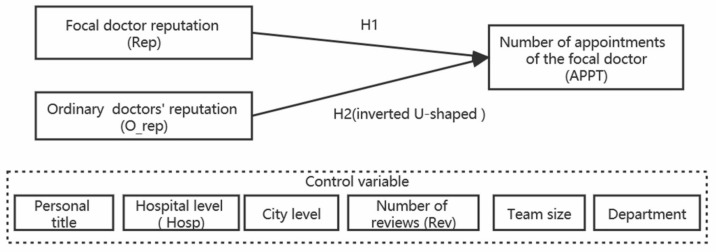
Conceptual model of leader.

The leader’s conceptual model is shown in Figure 1.

Reputation is formed by patients’ feedback on a doctor’s service quality, so it is a kind of information including doctor service quality. Online health platforms are set with scoring, likes, comments, and other functions so that patients who have received online medical services can give feedback on the services they have received. This kind of feedback may include a doctor’s medical level and service attitude. Online medical services are a kind of trust product. Patients have no accurate judgment on the service quality of doctors in advance. Users’ online feedback can alleviate information asymmetry and increase patients’ trust in doctors. Patients’ satisfaction with mobile Internet services and willingness to continue using mobile Internet services have a significant positive impact on word of mouth [20]. A good reputation is usually obtained by a high level of diagnosis and treatment and a positive service attitude [34]. Therefore, a doctor’s reputation is usually positively related to his or her service quality (diagnosis and treatment level and service attitude). So, having a high reputation can increase the chance of being selected by patients.

**Hypothesis** **1.** *There is a positive correlation between the focal doctor’s reputation and appointment numbers*.

In a team, leaders and other team members have similar goals and means. They all compete for a patient’s choice by displaying online information in the online medical service platform. Therefore, there is a dependency between the leader and the rest of the team. This dependence may be positive or negative. For the leader, the impact of the reputation of ordinary doctors in his or her team on his or her number of appointments may be positive or negative. On the one hand, ordinary doctors on the team belong to the same team as the leader. Their reputation together forms the reputation of the whole team. Improving the reputation of the team can also increase the chance that the leader is selected. At this time, he or she and ordinary doctors on the team form a cooperative relationship. On the other hand, when the reputations of ordinary doctors on the team are high enough, it shows that, in addition to the leader, there are many ordinary doctors with high service quality on the team for patients to choose from. At this point, there is a competitive relationship between the leader and the rest of the team. At this time, improving the reputation of ordinary doctors is detrimental to the number of appointments of the leader. Therefore, this paper believes that there is a theoretical basis for an inverted U-shaped relationship between ordinary doctors and leaders. Over time, we assume that the number of appointments for leaders will decrease as the reputation of ordinary doctors on the team increases to a certain value. Therefore, we assumed the following:

**Hypothesis** **2.** *There is a nonlinear relationship (inverted U-shaped relationship) between the ordinary doctors’ reputations on the team and the focal doctor’s appointment numbers*.

#### 2.1.2. Hypotheses of Ordinary Doctors

**Figure 2:**
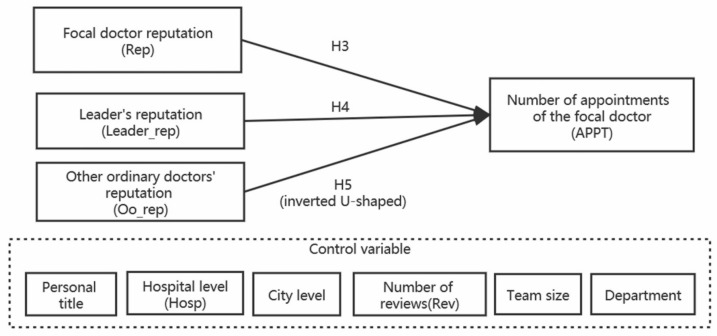
Conceptual model of ordinary doctor.

The ordinary doctor’s conceptual model is shown in Figure 2.

The patient’s feedback to the service of the ordinary doctor includes the service quality, service attitude, and other information in the online medical process of the ordinary doctor. During the appointment process, the patient can increase the understanding and trust of the ordinary doctor through his or her reputation. Therefore, we proposed the following assumptions:

**Hypothesis** **3.** *There is a positive correlation between the focal doctor’s reputation and appointment numbers*.

In our research, most of the leaders are well-known experts from high-level hospitals that have formed a brand effect. Leader reputation has a positive effect on team performance [35]. That is to say, the reputation of the team leader has a positive spillover effect on the number of appointments of the ordinary doctor. When patients make a choice among these doctors, the reputation of the leader can be transferred to the quality assessment of ordinary doctors. The reputation of the leader is equivalent to the brand of the team, which can attract patients to choose the team and then increase the chance of the ordinary doctor of the team to be selected. To this end, we proposed the following assumptions:

**Hypothesis** **4.** *The reputation of the team’s leader has a positive impact on the focal doctor’s appointment numbers*.

In a medical team, doctors have similar goals and means, so they are dependent on each other. Therefore, the reputation of other ordinary doctors on the team will have a spillover effect on their own performance, which may be positive or negative. On the one hand, because they belong to the same team, their reputations add up to form the reputation of the whole team. At this time, the team doctors form a cooperative relationship. On the other hand, when the reputation of other ordinary doctors on the team is high enough, there is a competitive relationship between focal doctor and other ordinary doctors on the team. This paper argues that there is a theoretical basis for the inverted U-shaped relationship between other ordinary doctors on the team and the appointment quantity of the focal doctor. Therefore, we assumed the following:

**Hypothesis** **5:** *There is a nonlinear relationship (inverted U-shaped relationship) between other ordinary doctors’ reputations on the team and the focal doctor’s appointment numbers*.

### 2.2. Research Methodology

#### 2.2.1. Variables’ Design 

When the leader is the focal doctor, the dependent variable is his/her cumulative appointment numbers (APPT) and the independent variable is his/her own reputation (Rep) and ordinary doctors’ average reputations on the team (O_rep). When the ordinary doctor is the focal doctor, the dependent variable is his/her cumulative appointment numbers (APPT) and the independent variable is his/her own reputation (Rep), leader’s reputation (Leader_rep), and other ordinary doctors’ average reputations on the team (Oo_rep).

Reputation was measured by two indicators: doctors’ likes and scores. The patient can like and rate the doctor on the doctor’s homepage after being served. Since these two variables may have different ranges, they are standardized and then averaged to create a composite variable.

Control variables at the individual level include the title, hospital level, city level, department, and review numbers and at the team level include team size. Table 1 and Table 2 are variables’ description of leaders and ordinary doctors.

#### 2.2.2. Empirical Model and Test Method

For the leader:

(1) $$\mathrm{APPT}\;=\;\beta_{0}+\;\beta_{1}\mathrm{Title}\;+\;\;\beta_{2}\;\mathrm{HospitalLevel}\;+\;\beta_{3}\mathrm{CityLevel}\;+\;\beta_{4}\mathrm{ReviewNumber}\;\;+\;\beta_{5}\mathrm{TeamSize}\;+\;\beta_{6}\mathrm{Reputation}\;+\;\beta_{7}O\_\mathrm{rep}\;+\beta_{8}\mathrm{Reputation}*\;O\_\mathrm{rep}\;+\;\mathrm{Department}\;\mathrm{dummies}+\;\varepsilon ,$$

For the ordinary doctor:

(2) $$\mathrm{APPT}\;=\;\beta_{0}\;+\;\beta_{1}\mathrm{Title}\;+\;\beta_{2}\;\mathrm{HospitalLevel}\;+\;\beta_{3}\mathrm{CityLevel}\;+\;\beta_{4}\;\mathrm{ReviewNumber}\;+\;\beta_{5}\;\;\mathrm{TeamSize}\;+\;\beta_{6}\;\mathrm{Reputation}\;+\;\beta_{7}\;\mathrm{Leader}\_\mathrm{Rep}\;+\;\beta_{8}\;\mathrm{Oo}\_\mathrm{rep}\;+\;\beta_{9}\;\mathrm{Reputation}*\;\;\mathrm{Leader}\_\mathrm{rep}\;+\;\beta_{10}\;\mathrm{Reputation}*\;\mathrm{Oo}\_\mathrm{rep}\;+\;\beta_{11}\;\mathrm{Leader}\_\mathrm{Rep}\;*\;\mathrm{Oo}\_\mathrm{rep}\;+\;\mathrm{Department}\;\mathrm{dummies}\;+\;\varepsilon ,$$

In most empirical identification of U-shaped relationships, researchers will include a nonlinear (usually quadratic) term in a standard linear regression model [36,37,38]. If this term is significant, and the estimated extreme point is within the data range, it is considered that there is a U-shaped relationship. Therefore, we add the quadratic term to the empirical model to perform a standard regression analysis to test whether the quadratic term is significant to test whether the extreme points are within the data range.

However, Lind and Mehlum demonstrated that this standard is too weak [39]. When the true relationship is convex and monotonous, the model estimate will erroneously produce an extreme point and a U-shaped relationship. Lind and Mehlum used the general framework developed by Sasabuschi to test whether there is a U-shaped and inverted U-shaped relationship between two variables, and used this test principle to write the utest test command [39]. Utest provides an “exact test” for the existence of a U-shaped (or inverted U-shaped) relationship in an interval. Therefore, we continued to use the utest command to perform an inverted U test of our empirical model.

## 3. Results

### 3.1. Results for Leaders

#### 3.1.1. Descriptive Statistic and Correlation for Leaders

The descriptive statistics and correlations for the key variables used in leader’s research model are presented in Table 3 and Table 4. 

#### 3.1.2. Regression Results of Leader’s Research Model 

Linear regression and hierarchical multiple regressions were used to estimate empirical results, while statistical significance was established at a *p*-value less than 0.05. All data were analyzed using STATA.

The regression results for the team leader are shown in Table 5. At this time, ordinary doctors on the team refer to doctors other than the leader on the team. From Model 2, it is noted that Rep (β = 0.0495, *p* > 0.1) has no effect on APPT. This may be because most leaders have busy offline tasks and limited online appointment opportunities. Therefore, their appointment numbers will not increase with his/her reputation’s improvement. H1 is not supported. (**H1:** *There is a positive correlation between the focal doctor’s reputation and appointment numbers***.**)

The coefficient of O_rep ‘s quadratic term (β = −0.0363 ***, *p* < 0.01) is negative and significant. This implies that the relationship between O_rep and APPT is nonlinear. Ordinary doctors’ reputations have an inverted U relationship with the focal doctor’s appointment numbers on the team, indicating that H2 is confirmed and in line with the social Interdependence theory (**H2:** *There is a nonlinear relationship (inverted U-shaped relationship) between the ordinary doctors’ reputations on the team and the focal doctor’s appointment numbers.*).

We used the utest command to test the inverted U-shaped curves in the empirical models.

For the leader’s model, we tested the inverted U-shaped relationship between O_rep and APPT, and we saw that the extreme point was 6.477 and the range of O_rep was [−0.489, 20.557]. It can be seen that the extreme point is within the data range. *p* >|t| = 0.00802, the result is significant at a statistical level of 5%, which proves that there is an inverted U-shaped relationship between O_rep and APPT. The utest results for leader doctors are shown in Table 6. 

### 3.2. Results for Ordinary Doctors

#### 3.2.1. Descriptive Statistic and Correlation for Ordinary Doctors

The descriptive statistics and correlations for the key variables used in ordinary doctors’ research model are presented in Table 7 and Table 8. 

#### 3.2.2. Regression Results of Ordinary Doctors’ Research Model

The regression results for ordinary doctors are shown in Table 9. From Model 4, it is noted that Rep (β = 0.329, *p* < 0.01) and Leader_rep (β = 0.318, *p* < 0.01) have a positive effect on APPT. Therefore, H3 and H4 are supported (**H3:** *There is a positive correlation between the focal doctor’s reputation and appointment numbers. ***H4:** *The reputation of the team’s leader has a positive impact on the focal doctor’s appointment numbers.*).

Further, we note that the coefficient of Oo_rep’s quadratic term (β = −0.0198, *p* < 0.01) is negative and significant. It means that the relationship between Oo_rep and APPT is nonlinear. Oo_rep has an inverted-U relationship with focal doctor’s appointment numbers on the team, indicating that H5 is confirmed and in line with the social Interdependence theory (**H5:** *There is a nonlinear relationship (inverted U-shaped relationship) between other ordinary doctors’ reputations on the team and the focal doctor’s appointment numbers.*).

We used the utest command to test the inverted U-shaped curves in the empirical models.

For the ordinary doctor’s model, we tested the inverted U-shaped relationship between Oo_rep and APPT, and we can see that the extreme point is 12.381 and the range of Oo_rep is [–11.467, 34.380]. It can be seen that the extreme point is within the data range. *p* >|t| = 0.000262, the result is significant at a statistical level of 5%, which proves that there is an inverted U-shaped relationship between Oo_rep and APPT. The utest results for leader doctors are shown in Table 10. 

## 4. Discussion

In this paper, the doctors in an online medical team were divided into the leaders and ordinary doctors. When the leader was the focal doctor, we studied the influence of the reputation of the focus doctor and the reputation of ordinary doctors on the team on the number of appointments of the focal doctor. The main findings were as follows.

The focal doctor’s reputation has no impact on his or her own appointment numbers, which means that his or her appointment numbers will not change due to his or her reputation. The leader doctor who takes the lead in setting up the online medical team is ordinarily a senior expert with rich experience in diagnosis and treatment and high social status. When a patient makes an appointment, the reputation of the leader doctor is not the main consideration of the patient. Another possible explanation is that the leader doctor is usually a senior expert, with busy offline tasks and limited appointment opportunities on the Internet. Due to the ceiling effect, the number of online appointments of the leader doctor will not increase with the improvement of senior reputation.

The relationship between the reputation of ordinary doctors on the team and the focal doctor’s appointment numbers is nonlinear, showing an inverted U-shaped relationship. This means that when the reputations of ordinary doctors on the team are at a low level, the reputations of ordinary doctors on the team have a positive spillover effect on the number of appointments of the focal doctor. At this time, these doctors cannot compete with the focal doctor. The higher the reputation of ordinary doctors on the team, the higher the focal doctor’s appointment numbers, and the cooperative relationship between them; when the reputations of ordinary doctors on the team are high, the reputations of ordinary doctors on the team have a negative spillover effect on the focal doctor’s appointment numbers. A high reputation represents a high quality of service. At this time, the service quality of ordinary doctors is also very high. They form a competitive relationship with the focal doctor.

When the ordinary doctor was the focal doctor, we studied the influence of his/her reputations, his/her leader’s reputation, and other ordinary doctors’ reputations on his/her number of appointments. The main findings are as follows.

The focal doctor’s reputation has a positive impact on his/her own appointment numbers. This means that the number of appointments for ordinary doctors increases with their reputation. For ordinary doctors, online reputation reflects their service quality. The higher the online reputation is, the higher the service quality is and the chance of being selected by patients increases. As a result, the number of appointments for ordinary doctors will increase with their reputations.

The reputation of the leader doctor has a positive impact on the focal doctor’s appointment numbers. This means that in an online health care team, the higher the reputation of the leader doctor, the higher the focal doctor’s appointment numbers. As the brand of the team, the leader doctor has a greater visibility impact. The higher his or her reputation is, the more opportunities his/her team’s doctors have to be selected.

There is a nonlinear relationship (inverted U-shaped relationship) between the reputation of other ordinary doctors on the team and the number of appointments of the focal doctor on the team. This means that when the reputations of other ordinary doctors on the team are at a low level, the reputations of other ordinary doctors on the team have a positive spillover effect on the number of appointments of the focal doctor. At this time, other ordinary doctors on the team are not enough to pose a competitive threat to the focal doctor; when the reputations of other ordinary doctors on the team are high, the reputations of other ordinary doctors on the team have a negative spillover effect on the number of appointments of the focal doctor. At this time, other ordinary doctors on the team compete with the focal doctor.

## 5. Conclusions 

### 5.1. Key Findings

There were several major findings in this study. For a leader doctor, his/her appointment numbers are not related to his/her own reputation, and there was an inverted U-shaped relationship with the ordinary doctors’ reputations on the team. For an ordinary doctor, his/her appointment numbers were positively correlated with his/her own reputation and positively correlated with his/her leader’s reputation and there was an inverted U-shaped relationship with the other ordinary doctors’ reputations. The research shows that there is a positive spillover effect on the team leaders’ reputation. There are two relationships between team doctors: competition and cooperation. This study provides guidance for the leader to select team members and the ordinary doctor to select a team.

### 5.2. Implications for Research

Online medical team is a new model of online health care. It is important to help hierarchical treatment, balance medical resources, and improve medical efficiency. Existing research has focused on the overall performance of the team [1,27]. However, there are few studies on team member relationships in this field. This study examined the impact of teammates’ online reputations on physicians’ online appointment numbers. It is important not only for doctors but also for platform providers of electronic consultation websites to understand the factors that affect doctors’ online reputations because it is a goal of platform providers to stimulate doctors to continue to participate and provide services.

Second, our research adds new insights to existing reputation theories. Previous research on online market reputation has focused on the analysis of individuals. Our research reveals the importance of member interaction. Our results provide evidence that teammates’ online reputations affect the focal doctor’s appointment numbers. This result suggests that when we study individuals in a team, we should bring the individual into the organizational environment and consider the influence of other team members.

This study expands and enriches the scope and meaning of the application of social interdependence theory. Social interdependence theory is mainly applied in the fields of psychology, education, and physical education. This study extends the social interdependence theory to the online health. This study explored the influence of the teammates’ reputations on the focal doctor’s appointment numbers. We found that there are two types of dependence between the leader doctor and the general doctor and between the general doctor and the general doctor, namely competition and cooperation.

### 5.3. Practical Significance

For the leader doctor, the results show that the leader doctor’s own reputation has no effect on the number of appointments. This suggests that the leader doctor does not need to spend too much effort on reputation building. An increase in the reputation of an ordinary doctor whose reputation is in the lower range has a positive spillover effect on the leader doctor’s appointment numbers. An increase in the reputation of an ordinary doctor whose reputation is in the higher range has a negative spillover effect on the leader doctor’s appointment numbers. Therefore, the leader doctor may select the ordinary doctor with a medium reputation as his/her member. 

For ordinary doctors, both their own reputations and the leader doctors’ reputation have a positive impact on their appointment numbers. Therefore, ordinary doctors can concentrate on improving their service quality to help improve their reputation. They can also choose to join a team of the leader with a high reputation. There is a nonlinear relationship (inverted U-shaped relationship) between the reputation of other ordinary doctors on the team and the focal doctor’s appointment numbers. It shows that when the reputations of other ordinary doctors on the team are at a low level, there is a cooperative relationship between them. When the reputations of other ordinary doctors on the team are at a high level, there is a competitive relationship. Therefore, a team with low reputations among other ordinary doctors on the team should be selected to join.

For designers and providers of online health platforms, this research helps them retain doctors and promote the prosperity of the platform. The results can help designers and providers of online health platforms understand how to improve the performance of doctors, especially how to help specific groups of doctors improve their performance. For example, a recommendation mechanism can be set up to recommend young doctors and general doctors with low reputations to the leader doctor as team members. It can also recommend online medical teams for young doctors and general doctors based on the reputation of the leader doctor and the overall reputations of the team’s ordinary doctors.

## 6. Limitations and Future Work

(1) A multilevel approach was not possible due to a lack of data on the team level. Since the website currently does not have team-level data, we were unable to design team-level variables. We believe that the data will be enriched with the improvement of website. Once the team-level data are available, we will consider further adding the team-level variables to our study for multilevel analysis.

(2) This study examined the effect of teammates’ reputations on the focal doctor’s appointment numbers. Since other attributes of teammates may also have an effect on the focal doctor’s appointment numbers, such as teammates’ effort and status. In a future study, we intend to examine the effect of other attributes of teammates, such as teammates’ effort and status, on the focal doctor’s appointment numbers. This will further enrich the social interdependence theory.

(3) This study examined only Guahao.com’s team of experts. Future research could be extended to medical teams or organizations on other online platforms. For example, China’s haodf.com has established a similar online medical team. To test whether our findings are still valid in other circumstances, we considered collecting data from other online health care platforms for future studies.

(4) Due to data limitations, cross-sectional data were used in this study. It is possible that some of the independent variables relevant to our work may have been missed. The panel data can effectively reduce or eliminate the impact of this problem. Therefore, the panel data will be considered for further research.

## Figures and Tables

**Table 1:** Variables’ description of leaders.

Variables	Variables Symbol	Explanation
Dependent variable	APPT	The cumulative number of patients who have made a appointment with the focal doctor.
Independent	Rep	Reputation of the focal doctor was obtained by averaging the number of likes and score of the focal doctor after standardization.
variables	O_rep	The average reputation of other doctors in the team except the focal doctor, that is, the average reputation of all ordinary doctors. It is calculated as follows. For each team, we first sum up the reputation of all members of the team, then subtract the reputation of the focal doctor, and divide the value obtained by the number of team members minus 1.
	O_rep2	The square of the average reputation of all ordinary doctors on the team.
Control variables	Title	1 to 5, with 1 being the lowest and 5 being the highest.
	City	1 to 7, with 1 being the lowest and 7 the highest.
	Hosp	1 to 4, with 1 being the lowest and 4 the highest.
	Rev	The number of reviews received by the focal doctor.
	TeamSize	The number of people of the team.

Notes: APPT = number of appointments; Rep = online reputation; O_rep = the average reputation of all ordinary doctors in the team; O_rep2 = The square of the average reputation of all ordinary doctors on the team; City = city level; Hosp = hospital level; Rev = review number; TeamSize = team size.

**Table 2:** Variables’ description of ordinary doctors.

Variables	Variables Symbol	Explanation
Dependent variable	APPT	The cumulative number of patients who have made an appointment with the focal doctor.
	Rep	Reputation of the focal doctor was obtained by averaging the number of likes and score of the focal doctor after standardization.
Independent	Leader_rep	The reputation of the leader physician on the team.
variables	Oo_rep	The average reputation of other doctors in the team except the focal doctor and the leader doctor, that is, the average reputation of other ordinary doctors except the focal doctor. For each team, we first sum up the reputation of all members of the team, then subtract the reputation of the focal doctor and the reputation of the leader doctor, and divide the value obtained by the number of people in the team minus the value after 2.
	Oo_rep2	The square of the average reputation of other ordinary doctors on the team.
Control variables	Title	1 to 5, with 1 being the lowest and 5 being the highest.
	City	1 to 7, with 1 being the lowest and 7 the highest.
	Hosp	1 to 4, with 1 being the lowest and 4 the highest.
	Rev	The number of reviews received by the focal doctor.
	TeamSize	The number of people of the team.

Notes: APPT = number of appointments; Rep = online reputation; Oo_rep = the average reputation of other ordinary doctors in the team; Oo_rep2 = The square of the average reputation of other ordinary doctors on the team; City = city level; Hosp = hospital level; Rev = review number; TeamSize = team size.

**Table 3:** Description statistics of leaders.

Variable	Obs	Mean	Std. Dev.	Min	Max
APPT	6103	543.64	2538.225	0.00	63,812
Rep	6103	0.196	1.072	−0.353	25.498
O_rep	6103	−0.094	0.831	−0.489	20.558
O_rep2	6103	0.699	6.849	0.00	422.621
Rev	6103	54.337	267.715	0.00	11,328
TeamSize	6103	5.107	3.025	1	88
Title	6103	4.774	0.433	1	5
Hosp	6103	2.978	0.164	1	3
City	6103	5.397	1.244	1	7

Note: APPT = number of appointments; Rep = online reputation; O_rep = the average reputation of all ordinary doctors in the team; O_rep2 = The square of the average reputation of all ordinary doctors on the team; City = city level; Hosp = hospital level; Rev = review number; TeamSize = team size, Obs = observation, Std. Dev. = Standard Deviation, Min = minimum, Max= maximum.

**Table 4:** Matrix of correlations of leaders.

Variables	(1)	(2)	(3)	(4)	(5)	(6)	(7)	(8)	(9)
(1) APPT	1.000								
(2) Rep	0.515	1.000							
(3) O_rep	0.377	0.374	1.000						
(4) O_rep2	0.114	0.073	0.672	1.000					
(5) Rev	0.810	0.543	0.346	0.120	1.000				
(6) TeamSize	0.082	0.159	0.047	−0.014	0.103	1.000			
(7) Title	0.048	0.073	0.052	0.010	0.025	0.112	1.000		
(8) Hosp	0.022	0.023	0.028	0.009	0.015	0.002	0.053	1.000	
(9) City	0.197	0.270	0.227	0.026	0.177	0.022	0.026	−0.038	1.000

Note: APPT = number of appointments; Rep = online reputation; O_rep = the average reputation of all ordinary doctors in the team; O_rep2 = The square of the average reputation of all ordinary doctors on the team; City = city level; Hosp = hospital level; Rev = review number; TeamSize = team size.

**Table 5:** Results of regression analyses of leaders.

	(1)	(2)
	model1	model2
Title	0.233 ***	0.218 ***
	(0.0591)	(0.0589)
City	0.328 ***	0.306 ***
	(0.0199)	(0.0198)
Hosp	0.212	0.178
	(0.146)	(0.142)
lnRev	0.992 ***	0.898 ***
	(0.0178)	(0.0304)
lnTeamSize	0.167 **	0.164 **
	(0.0804)	(0.0802)
Rep		0.0455
		(0.0527)
O_rep		0.472 ***
		(0.0689)
O_rep2		−0.0364 ***
		(0.0115)
_cons	−3.326 ***	−2.825 ***
	(0.529)	(0.521)
N	6103	6103
R2	0.552	0.559

Note: The numbers in the tables are mostly beta-values. The numbers in brackets are the standard errors. Department virtual variables are included in the model but are not shown in the table.; ** *p* < 0.01, *** *p* < 0.001; APPT = number of appointments; Rep = online reputation; O_rep = the average reputation of all ordinary doctors in the team; O_rep2 = The square of the average reputation of all ordinary doctors on the team; City = city level; Hosp = hospital level; Rev = review number; TeamSize = team size.

**Table 6:** U–test results of leader.

Dependent Variable: APPT		
Test: H1: Inverse U Shape vs. H0: Monotone or U Shape		
Specification: f(x) = x^2 Extreme Point: 6.477339		
	Lower bound	Upper bound
Interval	−0.4888113	20.55774
Slope	0.507623	−1.026038
*t*-value	6.543666	−2.408667
*p* > |*t*|	3.25 × 10^-11^	0.0080203
Overall test of presence of a Inverse U shape: *t*-value = 2.4 *p* > |*t*| = 0.00802		
95% Fieller interval for extreme point: [4.5529598; 13.757446]		

**Table 7:** Description statistics of ordinary doctors.

Variable	Obs	Mean	Std. Dev.	Min	Max
APPT	25,040	254.081	1,846.458	0	70,117
Rep	25,040	−0.048	0.713	−0.353	26.053
Leader rep	25,040	0.016	0.827	−0.479	13.106
Oo rep	25,040	0.027	0.815	−0.329	34.38
Oo rep2	25,040	0.666	12.253	0	1182.011
Rev	25,040	30.975	220.643	0	12,499
TeamSize	25,040	7.326	7.553	2	88
Title	25,040	3.678	0.915	1	5
Hosp	25,040	2.958	0.241	1	3
City	25,040	5.381	1.277	1	7

Notes: APPT = number of appointments; Rep = online reputation; Oo_rep = the average reputation of other ordinary doctors in the team; Oo_rep2 = The square of the average reputation of other ordinary doctors on the team; City = city level; Hosp = hospital level; Rev = review number; TeamSize = team size, Obs = observation, Std. Dev. = Standard Deviation, Min = minimum, Max= maximum.

**Table 8:** Matrix of correlations of ordinary doctors.

Variables	(1)	(2)	(3)	(4)	(5)	(6)	(7)	(8)	(9)	(10)
(1) APPT	1.000									
(2) Rep	0.497	1.000								
(3) Leader_rep	0.252	0.360	1.000							
(4) Oo_rep	0.301	0.358	0.318	1.000						
(5) Oo_rep2	0.058	0.040	0.026	0.658	1.000					
(6) Rev	0.716	0.530	0.217	0.244	0.048	1.000				
(7) TeamSize	0.015	0.031	0.296	0.004	−0.015	0.018	1.000			
(8) Title	0.110	0.184	−0.001	0.061	0.002	0.057	−0.062	1.000		
(9) Hosp	0.023	0.038	−0.115	0.002	0.003	0.004	−0.243	0.090	1.000	
(10) City	0.138	0.247	0.237	0.197	0.010	0.113	−0.088	0.070	−0.023	1.000

Notes: APPT = number of appointments; Rep = online reputation; Oo_rep = the average reputation of other ordinary doctors in the team; Oo_rep2 = The square of the average reputation of other ordinary doctors on the team; City = city level; Hosp = hospital level; Rev = review number; TeamSize = team size.

**Table 9:** Results of regression analyses of ordinary doctors.

	(3)	(4)
	Model3	Model4
Title	0.493 ***	0.512 ***
	(0.0118)	(0.0114)
City	0.234 ***	0.175 ***
	(0.00751)	(0.00727)
Hosp	0.216 ***	0.319 ***
	(0.0327)	(0.0325)
lnRev	0.810 ***	0.548 ***
	(0.0120)	(0.0324)
lnTeamSize	0.113 ***	−0.0153
	(0.0229)	(0.0230)
Rep		0.334 ***
		(0.0836)
Leader_rep		0.316 ***
		(0.0243)
Oo_rep		0.481 ***
		(0.0364)
Oo_rep2		−0.0194 ***
		(0.00396)
_cons	−3.560 ***	−3.116 ***
	(0.142)	(0.138)
N	25,040	25,002
R2	0.492	0.525

Note: The numbers in the tables are mostly beta values. The numbers in brackets are the standard errors. Department virtual variables are included in the model but are not shown in the table; *** *p* < 0.001.

**Table 10:** Utest results of ordinary doctors.

Dependent Variable: APPT		
**Test: H1: Inverse U shape vs. H0: Monotone or U Shape**		
**Specification: f(x) = x ^ 2** **Extreme Point: 12.38148**		
	Lower bound	Upper bound
Interval	−11.46711	34.38039
Slope	0.92726	−0.8553423
*t*-value	7.7152	−3.468163
*p* > |t|	6.25 × 10^−15^	0.0002625
Overall test of presence of a Inverse U shape: *t*-value = 3.47 *p* > |t| = 0.000262		
95% Fieller interval for extreme point: [9.6079864; 18.563818]		
